# An Intelligent Anomaly Detection Approach for Accurate and Reliable Weather Forecasting at IoT Edges: A Case Study

**DOI:** 10.3390/s23052426

**Published:** 2023-02-22

**Authors:** Şükrü Mustafa Kaya, Buket İşler, Adnan M. Abu-Mahfouz, Jawad Rasheed, Abdulaziz AlShammari

**Affiliations:** 1 Department of Computer Technologies, Istanbul Aydin University, Istanbul 34295, Turkey; 2 Department of Computer Engineering, Istanbul Topkapi University, Istanbul 34087, Turkey; 3 Council for Scientific and Industrial Research (CSIR), Pretoria 0184, South Africa; 4 Department of Electrical and Electronic Engineering Science, University of Johannesburg, Johannesburg 2006, South Africa; 5 Department of Software Engineering, Istanbul Nisantasi University, Istanbul 34398, Turkey; 6 College of Computer and Information Sciences, Imam Mohammad Ibn Saud Islamic University (IMSIU), Riyadh 11432, Saudi Arabia

**Keywords:** internet of things, edge computing, weather forecasting, data pre-processing

## Abstract

Industrialization and rapid urbanization in almost every country adversely affect many of our environmental values, such as our core ecosystem, regional climate differences and global diversity. The difficulties we encounter as a result of the rapid change we experience cause us to encounter many problems in our daily lives. The background of these problems is rapid digitalization and the lack of sufficient infrastructure to process and analyze very large volumes of data. Inaccurate, incomplete or irrelevant data produced in the IoT detection layer causes weather forecast reports to drift away from the concepts of accuracy and reliability, and as a result, activities based on weather forecasting are disrupted. A sophisticated and difficult talent, weather forecasting needs the observation and processing of enormous volumes of data. In addition, rapid urbanization, abrupt climate changes and mass digitization make it more difficult for the forecasts to be accurate and reliable. Increasing data density and rapid urbanization and digitalization make it difficult for the forecasts to be accurate and reliable. This situation prevents people from taking precautions against bad weather conditions in cities and rural areas and turns into a vital problem. In this study, an intelligent anomaly detection approach is presented to minimize the weather forecasting problems that arise as a result of rapid urbanization and mass digitalization. The proposed solutions cover data processing at the edge of the IoT and include filtering out the missing, unnecessary or anomaly data that prevent the predictions from being more accurate and reliable from the data obtained through the sensors. Anomaly detection metrics of five different machine learning (ML) algorithms, including support vector classifier (SVC), Adaboost, logistic regression (LR), naive Bayes (NB) and random forest (RF), were also compared in the study. These algorithms were used to create a data stream using the time, temperature, pressure, humidity and other sensor-generated information.

## 1. Introduction

The earth’s surface cover, or land cover, changes over time as a result of both natural and human factors [1]. One example of a land cover altering as a result of natural processes, such as tsunamis, erosion, landslides and floods, is continental movements [2]. Urbanization is one of the major drivers behind human-induced land use/cover change. Land use changes occur constantly and at many scales and can have specific and cumulative effects on the climate [3]. One of the biggest ecological and social issues of the twenty-first century is climate change, which is crucial for the continuation of life. Increased heat waves, droughts and floods brought on by climate change have already gone beyond what plants and animals can withstand, resulting in widespread extinctions of species such as coral reefs and trees. As a result of these extreme weather events, millions of people have been exposed to acute food and water insecurity [4]. It is vital to be able to make accurate and reliable estimates to minimize these and similar negativities and to take effective measures.

Kulkarni and Mukhopadhyay state that weather forecasting is an important field of study in meteorology and is one of the constantly challenging problems around the world. In addition, they propose a method based on the management of remote weather forecasting processes by utilizing IoT environments and sensor technologies in their studies [5]. In this way, Pauzi and Hasan are creating an IoT-based weather report system to monitor weather report systems in real time. In their work, it is mentioned that machine-learning algorithms are more successful than traditional statistical methods and they are developing the IoT-based weather report system. In addition, they use three different sensors such as—humidity, temperature, and rain—as well as a software interface with an ESP32 microcontroller to develop the weather report system [6]. In another study, Rameshwaraiah et al. tested the classification success of algorithms using machine-learning techniques such as SVC, RF, NB, decision trees (DT), and deep neural networks (DDN) on the data flow created with the internet of things environments [7]. Chowdury et al. are developing an IoT-based real-time river water quality monitoring system as part of their research. They created a sensor-based system to check the quality of the water. The primary element of the study is wireless sensor networks, which produce information about water through the sensors they utilize in conjunction with the microcontroller and send that information to the appropriate people via SMS. The suggested approach is therefore expected to significantly aid Bangladeshi populations in becoming aware of dirty water and encouraging them to avoid polluting the water [8]. Loannou et al. provide a comprehensive review of existing technologies for the implementation of automatic weather stations. Also, the internet of things, edge computing, deep learning, LPWAN, etc., will be used in the implementation of future AWS-based observation systems. The use of current technologies is also included. Finally, the study presents a case study developed in AWS (AgroComp project) and its results [9]. The work of Kulkarni et al. is about the nature of a weather display method using low-cost components that even any electronics enthusiast could design. Instead of sensors, data from stations around the world are used, through a global data supplier, to collect weather-related data.

Weather events have many parameters that cannot be predicted and calculated. In addition, the development of communication methods and specialized systems for weather forecasting can share data, and thus hybrid systems, emerge. Despite all these developments, the extension systems developed for weather forecasting are not completely reliable [5]. The system proposed by Sivakumar and Nanjundaswamy is geared towards using IoT technology to make real-time data accessible over a wide range, and very easily. Using multiple systems, the system deals with monitoring weather and climate changes such as temperature, humidity, wind speed, humidity, light intensity, UV radiation and even carbon monoxide levels in the air. Sensors send the data they collect to a web page and this web page can be accessed from anywhere in the world. An API is used in the study to accurately predict complex weather events. This API can be used to transport and store data from one place to another. It is also less costly than those on the market and will reduce the costs of monitoring air systems. This project can be used for meteorological stations and departments, the maritime and aviation industries, and even the agricultural industry [10]. An IoT-based weather monitoring and reporting system are being developed in a different project. In the study, data on temperature and humidity are automatically gathered and saved in a database to compare the old data with the new data.

The study provides the opportunity to predict the problems that may be encountered in the IoT systems used in the measurement stations established for weather forecasting. After data preparation and analysis, IoT makes data accurate and trustworthy. The study’s findings indicate that choosing the appropriate methodologies for anomaly detection at IoT edges and key elements such as volume, speed, diversity, accuracy and value—which are essential in real-time IoT systems—will be made possible. Another contribution of the study is to make the data produced by the sensors more meaningful and manageable by detecting anomalies between the IoT edges and the detection layer and the network layer when compared with the studies on weather forecasting and climate change. In addition, by doing this, the negative effects of anomaly data will be minimized for service providers, users and other interested parties.

As a result, the structure of the article is as follows. In Section 2, details are provided on the IoT system layers that enable the prediction of potential issues at the measurement stations set up for weather forecasting. In Section 3 Weather forecast and its importance are explained in detail. The difficulties encountered in weather forecasting are explained. Section 4 explains what types of tools are used to obtain the data used in the analysis. Results are delivered in Section 5, giving a comparison which was obtained through both experiments and simulations. Finally, Section 6 and Section 7 present a discussion and conclusion.

## 2. IoT and Edge Computing

**Figure 1:**
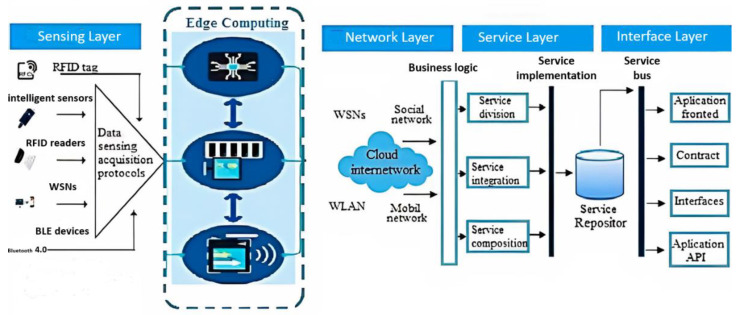
Intelligent Data Processing Layer [12]. Sensing Layer: To detect and gather information, this layer consists of sensing tools including smart sensors, RFID, and IoT client components. Edge (Pre-processing) Layer: The pre-processing (edge) layer, where the data detected by the sensors in the detection layer is filtered and cleaned from anomalies before exiting to the network layer. Network Layer: This is the layer that supports the Internet and other device connectivity infrastructure. Service Layer: The process of maintaining and offering services to users or other apps is visible at this layer. Interface Layer: This layer offers users or other services an interface [13].

Traditional IoT systems that have been developing in the last 25 years are generally designed on four layers. These layers are the sensing, network, service and interface application layers [11]. Many sensors such as humidity, temperature, pressure, movement, color and odor sensors are used to transfer the events that occur in the physical world to IoT systems. The sensing layer consisting of these sensors transfers all the events it detects to an IoT system. However, this layer of sensors is not suitable for high-performance data processing. Although there are alternatives with unlimited processing capacity such as the cloud, it is a big disadvantage that they are physically far from the sensing layer. Naturally, only cloud-based modeling is insufficient for an IoT system with high performance and real-time data processing. To eliminate these and similar problems and increase the high-performance data processing capacity, an edge computing mechanism working with IoT layers can be developed [12]. Figure 1 presents the edge computing layer where the sensor data produced in the meteorology domain will be processed, and the layers that make up the traditional IoT systems.

## 3. Weather Forecast and Its Importance

Since the 19th century, the basis of weather forecasting has been the examination of atmospheric variables, such as temperature, radiation, air pressure, wind speed, wind direction, humidity and precipitation. The process of finding trends in these data using various tools and algorithms is known as weather forecasting [14]. Innovating techniques including data assimilation, machine learning and ensemble modeling have been applied recently to enhance preexisting forecasting models [15]. Despite all these developments, many factors reduce the success of weather forecasting. Climate change is one of these factors. Deterioration of natural resources, destruction of vegetation, unplanned urbanization, increasing population density, and changing climates due to human activities, are signals for the future of humanity gives [16]. Extreme weather events (storms, heatwaves, droughts, floods, cyclones, etc.) caused by climate change negatively affect prediction success. Today, global warming is widely acknowledged as one of the greatest hazards to life and is no longer merely a theoretical concern. If recent trends in global warming persist, a rise in temperature, rising ocean levels, and an increase in the frequency of extreme weather events (storms, heatwaves, droughts, floods, cyclones, etc.) might result in catastrophic food shortages, home loss, and water shortages. In 2021, records for many extreme weather events were broken one after the other around the world. There have been disruptive weather-related incidents around the world (for example, Katrina and Rita in the USA). The Asian Continent was particularly prone to floods, typhoons and tsunamis. 1024 unusual meteorological phenomena were noted in Turkey last year, according to the Turkish State Meteorological Service’s forecast for 2021. The increase in extreme weather events observed in the last 20 years reached the highest level in 2021. There were 984 extreme weather events in 2020, while 935 events were recorded in 2019, including forest fires in Australia and USA, etc. Another four million people were left homeless. In addition, the monsoon disaster in Pakistan in June 2022, which cost about 1600 lives and made another four million people homeless, serves as a somber reminder of the negative environmental effects of global warming. Weather forecasting is a vital process that predicts and reduces the damage caused by disasters before they occur. Accurate forecasts made days or weeks in advance are an important factor in increasing economic efficiency. In addition, it may affect a variety of industries, including agriculture, irrigation, and maritime trade, and it may prevent fatal accidents [17].

The second factor affecting weather forecasting success is that a significant amount of data must be evaluated, and this raw data reveals systemic mistakes that must be fixed using statistical post-processing techniques [18]. Big data analytics tools can be used to overcome this issue. As was previously said, given the continuously rising social and economic significance of numerical weather forecasting, it must be precise and calibrated, for instance in agriculture and the renewable energy sector. Big data analytics can be used to enhance weather forecasts [19].

## 4. Materials and Methods

This work focuses on anomaly identification on a data stream made up of four-dimensional temperature, humidity, pressure and time data to increase the accuracy and dependability of the data used in weather forecasting. The data stream received from the IoT sensing layer is simulated for anomaly sensing. In the simulation, anomaly detection is performed between the first and second layers of the traditional IoT. This preprocessing layer, which will undertake the mission of edge computing, helps to create more meaningful big data by supporting the concepts of accuracy and reliability. In the study, the platform we created to generate the data stream for anomaly detection is presented in Section 4.1.

Studies using various techniques to detect anomalies in real-time data flow have been published in the literature. The goal of this study is to identify inaccurate data in a data stream that includes time, temperature, humidity and pressure values for testing. Anomaly detection occurs between the detection layer and the network layer. The Adaboost algorithm is initially employed in the study to find anomalies, after which the classification accuracy and data processing time of the SVC, Adaboost, LR, NB, and RF algorithms used to detect anomalies are compared. IoT systems are composed of raw data collected from IoT devices. The vast majority of beneficial IoT services are developed by collecting and handling data from objects. Numerous wireless technologies, including wireless sensor networks (WSN), low power wide area networks (LPWAN), WiFi, Bluetooth and cellular networks, are used to collect data. The server is in charge of managing the network data. IoT devices send the data they produce to a cloud-based server. The server searches through the gathered data to get relevant data [20]. Of course, smart IoT services receive crucial data. Data integrity becomes a crucial component of data analysis when there is a lot of data concentrated on the server. Data integrity lowers the computing strain on the server as it examines the data received from the IoT device. As the server processes data, the processing load is reduced, which helps save energy. A system must therefore support data integrity.

**Figure 2:**
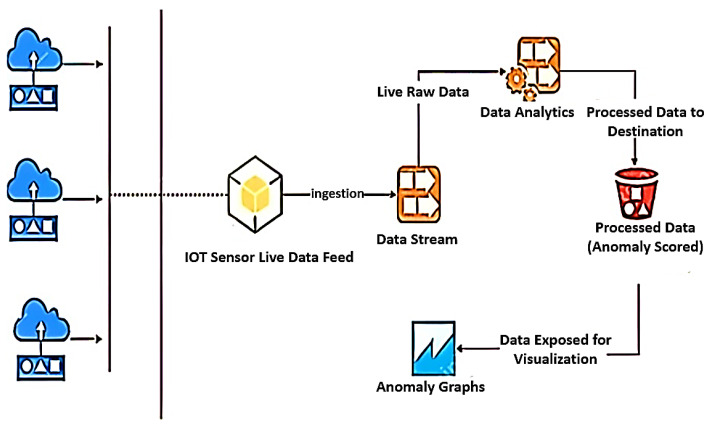
Architecture Used for Anomaly Detection and Performance Testing [13].

The architecture that can be employed for data integrity is shown in Figure 2. In IoT networks, several types of data are produced and sent to the cloud via gateways. Data is sent to the administration server in the cloud, which oversees the smart services. Servers examine the gathered data, providing useful data for intelligent services. Data integrity must be maintained for accurate data analysis. If data integrity is not ensured, the information that is extracted will not be trustworthy, and inaccurate information will result in poor decision-making. The suggested system is positioned in front of the server to support data integrity. Anomalies are found by conducting a real-time analysis of the data generated.

### 4.1. Case Study

**Figure 3:**
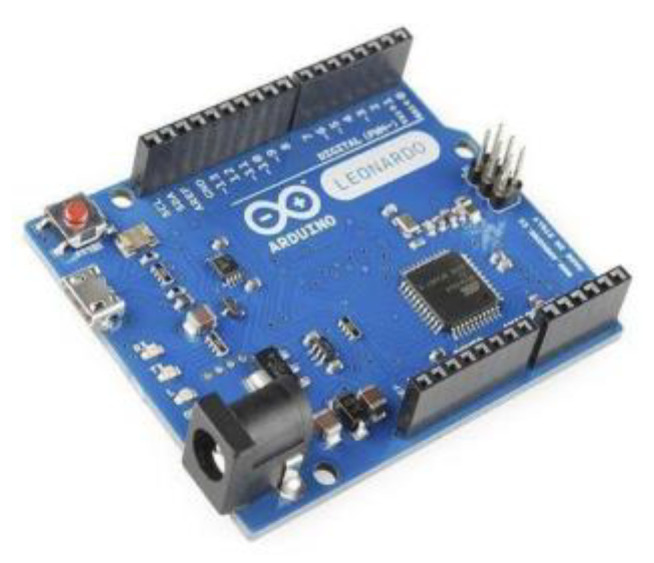
Arduino UNO R3 [21].

**Figure 4:**
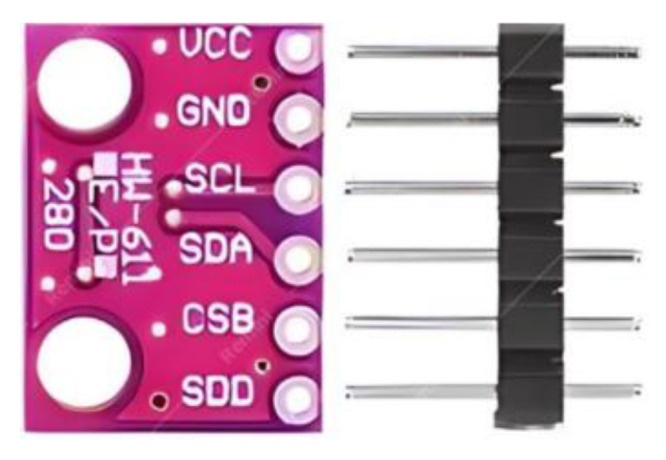
BME280 [22].

**Figure 5:**
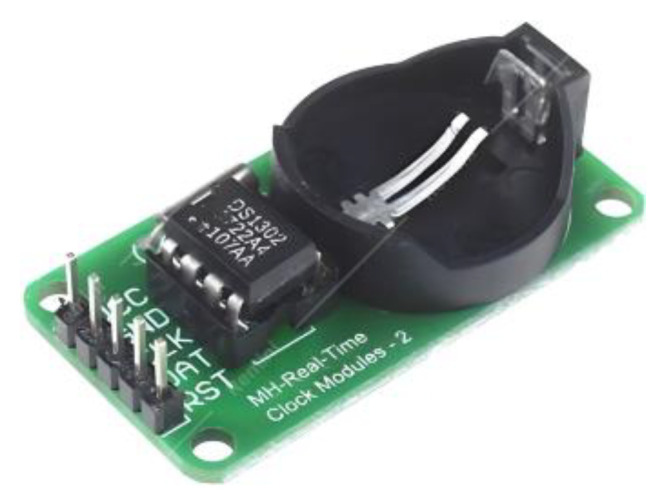
DS1302 Real Time Clock Module [23].

**Figure 6:**
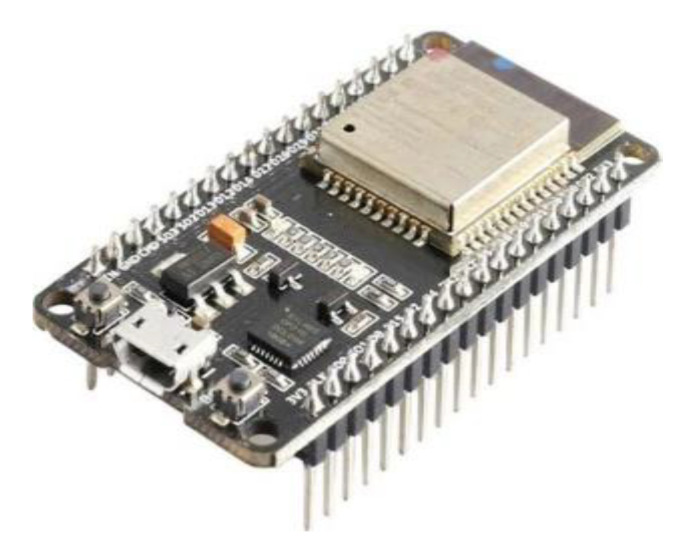
ESP32 Microcontroller [24].

In our study, the classification and anomaly detection success of SVC, Adaboost, LR, NB, and RF classifier algorithms were compared and presented. To create the data flow used in anomaly detection, Arduino cart, temperature, pressure, humidity sensors and a time regulator were used in the generation of raw data. While creating the platform, Arduino UNO R3 (Arduino, Turin, Italy) shown in Figure 3 and the BME280 sensor (Bosch Sensortec, Reutlingen, Germany) shown in Figure 4 were used to make temperature, pressure, and humidity measurements. In addition, the DS1302 clock module (Arduino) presented in Figure 5 was used to keep information about real-time while producing raw data. The ESP32 microcontroller (Espressif Systems, Shanghai, China) shown in Figure 6 was used for the capture and transfer process of the raw data obtained.

Arduino Uno microcontroller board, which is Atmega328-based, has a USB port, a power socket, an ICSP connector, six analog inputs, a 16 MHz crystal, fourteen digital input/output pins, six of which can be used as PWM outputs, and a reset button [21]. The Bosch BME280 was used in the study as a combined digital humidity, pressure and temperature sensor. It is based on tried-and-true sensing principles. The sensor module is housed in a compact metal lid LGA package with a 0.93 mm height and a 2.5 mm2 footprint. It monitors temperature with a precision of 1 °C, pressure with a precision of 1 hPa, and humidity with a precision of 3% [22].

Calendar/real-time clock chip DS1302 includes two power supplies, a big 2.5 V to 5.5 V working voltage range, and performs excellently. In addition, the chip has 31 bytes of static RAM. It communicates with a microprocessor using a simple serial interface. A real-time clock or calendar will display information such as seconds, minutes, hours, days, dates, months and years. It was used in the research to precisely record data on temperature, pressure and humidity [23]. The ESP32, a feature-rich microcontroller with an integrated Bluetooth and Wi-Fi connection for a variety of applications, is another project component. In addition, it has a structure that makes it easier to develop IoT applications. With a 40 °C to +125 °C operating temperature range, the ESP32 can function stably in industrial situations [24].

For real-time IoT designs, accuracy and speed measurements are two crucial elements. For this reason, Kim et al. recommend a classifier-based approach to data filtering for cloud servers. The developed data pre-processing method is used to pre-process the data before it is transmitted to the server. Initially, raw data is collected from the objects with the help of the sensing layer. After gathering the raw data, NB classifiers were used to determine whether the data were contaminated. The workload involved with processing data is reduced by sending the data to the server for analysis after pre-processing [25].

### 4.2. Data Set

The sensors operating in the IoT sensor layer in the study output 9690 data points consisting of four-dimensional temperature, humidity, pressure and time values. About 70% of the sensor data produced, or 6783 records, are utilized for training, and about 30% of the total data, or 2907 entries, are used for verification and prediction. In addition, modeling and normality operations are performed using the scikit-learn module.

**Figure 7:**
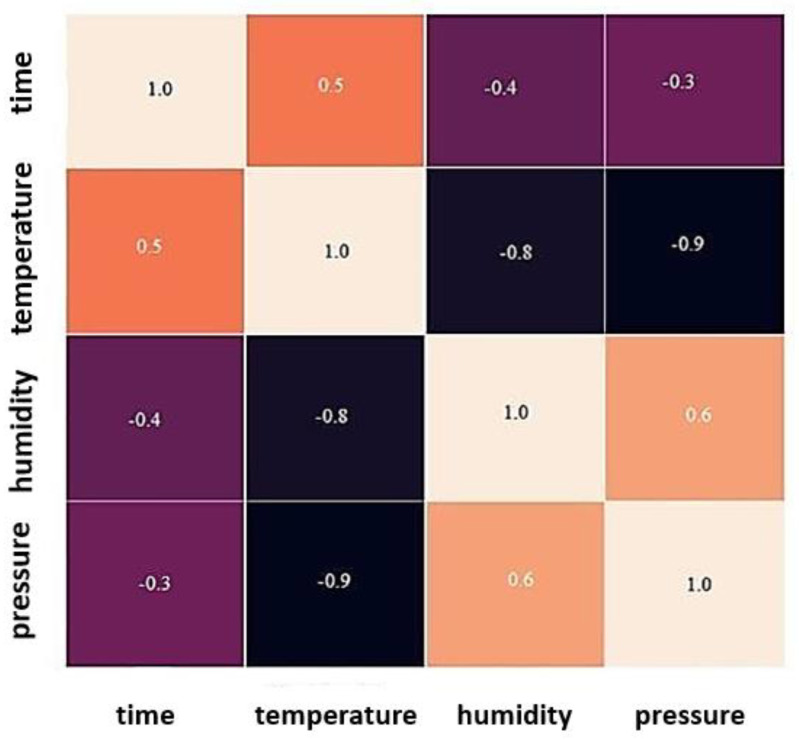
Correlation Matrix.

Exploratory data analysis was performed on the data. Within the scope of the analysis, temperature, humidity, pressure, and time attributes were examined among them. The correlation matrix was created and the features that had a high impact on the result were included in the training set. Figure 7 shows the correlation matrix.

As seen in the correlation matrix, there is a direct effect between pressure, humidity and temperature. A similar ratio appears between temperature and time. It is clear from the relationship between the data that the time and temperature measurements are better at spotting anomalies. The effects of temperature, humidity, and pressure are employed in our training set since they have similar effects on one another.

#### Simulation Model

**Figure 8:**
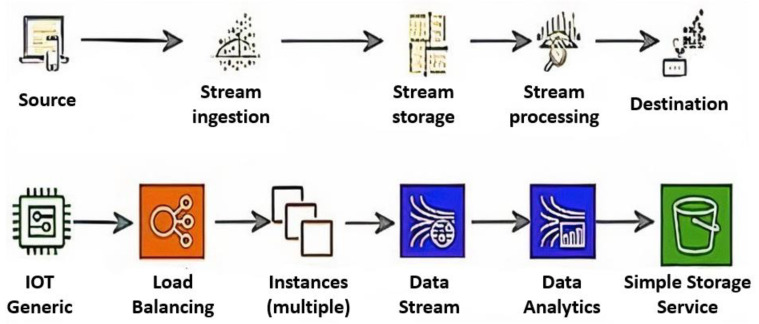
Simulation Model [13].

- Real-time data is received and processed simultaneously with the kinesis data analysis in the kinesis data stream, which will be utilized as the source. The results are then stored in the target region.
- The temperature sensor is classified as an internet of things (IoT) device to replicate data flow. The computed temperature values are accepted as standard data.
- All values outside the normal temperature values are determined as anomaly temperature values and filtered.
- To determine whether the data produced by the sensors is abnormal or normal, temperature values of 20 degrees and above measured between 24.00 and 07.00 are considered an anomaly; if the temperature between 12.00 and 15.00 is lower than 25 degrees, it is considered an anomaly. Algorithms are classified according to these criteria.
- 9690 data points, including temperature, humidity, pressure, and time values, were generated for the study utilizing an Arduino UNO R3, BME280, DS1302 real-time clock module and an ESP32 microcontroller. About 70% of the sensor data produced, or 6783 records, are utilized for training, and about 30% of the total data, or 2907 entries, are used for verification and prediction.
- The generated data is combined with Google Colab services to create a data stream. A total of 30% of the created data (or 2907 data) is used for prediction and verification, while the remaining 70% (or 6783 data) is used for instructional reasons. To maintain data integrity, after the anomalous data is discovered, dependable and accurate data is separated from the anomaly and transferred to the designated destination.
- Figure 8 shows the data stream simulation model developed to detect anomalies in temperature data with Adaboost, one of the machine-learning algorithms.

## 5. Experimental Results

### 5.1. Classification Reports

This study uses several metrics, such as precision, recall, accuracy, and F1-score, to evaluate the findings. A diagram of the confusion matrix is also included. The confusion matrix is a 2 × 2 matrix. The matrix contains four outputs that are *TP*, *TN*, *FP* and *FN* respectively. Metrics such as sensitivity, specificity, accuracy, and the error rate can be obtained using the confusion matrix [25].

As a result, attempts have been made to determine which algorithm does the best data preprocessing in weather forecasts according to the classification success and comparison matrices of the algorithms.

The confusion matrix produces the following results:

1. A group of data points that have been correctly identified as anomalies constitutes a true positive.
2. A handful of points that have been appropriately classified as normal are known as true negatives.
3. Few points that have been incorrectly classified as anomalies are known as false positives.
4. A handful of points that have been incorrectly classified as normal are known as false negatives [26].

Accuracy is a metric that is assessed in terms of the performance of the model as a whole. In the measurement of physical property, it is the discrepancy between the real value and the value displayed by the device. The accuracy estimate is represented by Equation (1):

(1) $$\mathrm{Accuracy}=\frac{TP+TN}{TP+TN+FP+FN}$$

Success is a circumstance that is as expected, as indicated by precision. It is a percentage of all genuine positives that the model claims are connected with all positives that the model demands. Using the Equations, a precision determination can be carried out (2).

(2) $$\mathrm{Precision}=\frac{TP}{TP+FP}$$

Another indicator, recall (also known as sensitivity), compares the total positives in system states with the precise number of positives in the data. Equation (3) presents the rate of recall.

(3) $$\mathrm{Recall}=\frac{TP}{TP+FN}$$

The F1-score, which served as the main study indicator, could also be used to calculate model performance. It is the harmonic mean of recall and precision. The F1-score shown in Equation (4) has the following value:

(4) $$\mathrm{F1-score}=\frac{2\times \left(\mathrm{Recall}\times \mathrm{Precision}\right)}{\left(\mathrm{Recall}+\mathrm{Precision}\right)}$$

The metrics of the data used for verification and prediction as well as the classification-related impact of the RF approach are displayed in Table 1 combined. The random forest algorithm was used with the grid search technique to determine the ideal settings. The maximum depth parameter is set to 4, the maximum features parameter to auto, and the maximum number of estimators to 200. Within the parameters of the classification criteria, gini is used to derive the criterion parameter.

The classification outcome of the LR algorithm is displayed in Table 2. The research shows how the metrics are affected by the data that makes up 30% of the data set.

The grid search based on the LR algorithm was used to find the optimal parameters. Within the parameters of the classification criterion, the C parameter was determined to be 0.1 and the penalty parameter to be l2.

Table 3 shows the classification result of the Adaboost algorithm. The result shows the impact of data used for validation and prediction, representing 30% of the data set, on metrics. The best parameters were determined by using the grid-search method on the Adaboost algorithm. Within the scope of classification criteria, the base estimator criterion parameter is gini, the base splitter parameter is best, and the n_estimators parameter is calculated as 1.

Table 4 shows the classification result of the NB algorithm. The measurements show the impact of the data used for validation and prediction, which accounts for 30% of the data set. The best parameters were determined by using the grid-search method over the NB algorithm. The var_smoothing parameter was calculated as 1.0 within the scope of the classification criteria.

The classification outcome of the SVC algorithm is displayed in Table 5. The measurements show the impact of the data used for validation and prediction, which accounts for 30% of the data set. The grid-search approach on the SVC algorithm was used to find the optimal parameters. The C parameter was computed to be 1.0, the gamma parameter to be 0.1, and the kernel parameter to be rbf within the parameters of the classification criterion.

Table 6 shows the accuracy scores of the classification algorithms. The results of accuracy scores are RF 1.0, LR 1.0, Adaboost 1.0, NB 0.95, and SVC 1.0.

RF, LR, Adaboost, NB, and SVC data processing speeds are shown in Table 7 in seconds, based on execution time.

### 5.2. Performance Curve

**Figure 9:**
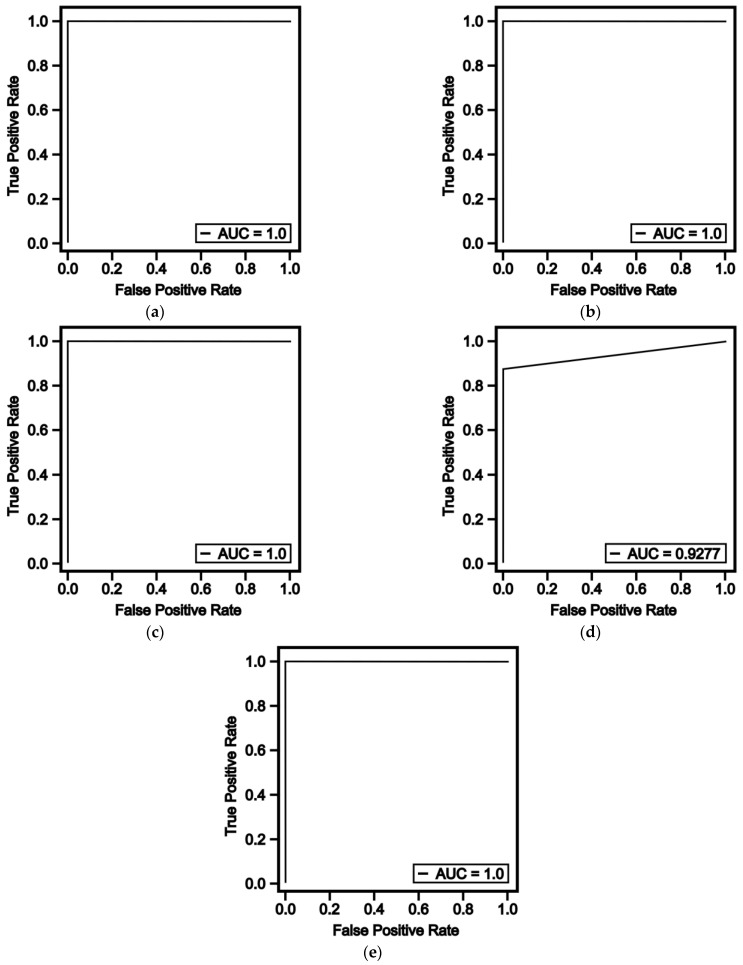
Comparison of ROC curves for (**a**) random forest, (**b**) logistic regression, (**c**) support vector machine, (**d**) Naïve Bayes and (**e**) Adaboost.

On a graph known as a ROC curve, a classification model’s performance is shown (receiver operating characteristic curve). The true positive rate (TPR) against the false positive rate (FPR) at various cut-off points of a parameter is plotted on the ROC curve. The FPR and TPR are shown on the x- and y-axes, respectively, to represent the ROC curve. This indicates that the test’s overall accuracy increases as the ROC curve approach the upper left corner. Which algorithms anticipate the best outcomes during the classification process are determined using the area under the curve (AUC) [26]. The performance curves of the ML algorithms we tested in the study are presented in Figure 9. The performance curves are examined, and it is seen that RF, LR, SVC and Adaboost algorithms make very close predictions to obtain almost the same AUC score, while the AUC score of the NB algorithm, unlike the others, remains at 0.92.

### 5.3. Confusion Matrix

The confusion matrix for the RF method is displayed in Table 8. When the table is studied, it becomes clear that 2907 data points, or 30% of the total data set, are employed in the calculation and verification of the numerical distribution. All of the data were appropriately categorized as a consequence of the analysis.

The confusion matrix values for the LR algorithm are displayed in Table 9. The distribution of 2907 data points from the data set used for the confusion matrix’s validation and prediction is depicted in the figure. 2031 of the data used for validation and prediction were each projected to be “true positives” by the algorithm, meaning that they were normal data with no anomalies. In addition, it accurately identified 876 data points as “true negatives”.

Table 10 shows the complexity matrix of the SVC. The algorithm does not misclassify any of the 2907 data used in validation and prediction.

Table 11 shows the NB algorithm’s confusion matrix. The table displays the distribution of 2907 data points for temperature, pressure, humidity and time under both actual and anticipated labels. The system predicts 1998 as normal data and a genuinely positive, not an anomaly, out of the 2907 normal data points used for verification and prediction. 112 typical data points were predicted by the algorithm to be anomalies and false negatives. The program successfully predicted true negatives and 764 data points with anomalies but predicted 34 data points with abnormalities as normal and false positives.

Table 12 shows the complexity matrix of the Adaboost. Of the 2907 data points used in verification and prediction, which are normal data, 2031 are predicted by the algorithm as normal data and true positive, with no anomaly. The algorithm accurately predicts true negatives, 876 data with anomalies. The trained Adaboost algorithm classifies the test set as perfect.

## 6. Discussion

The application of IoT technology to weather forecasting is looked at in this study. As can be observed, the systems created for weather forecasting make use of the traditional IoT architecture, which is made up of four layers: detection, network, service and application. The idea behind the proposed data pre-processing model is to reduce the issues that can arise in systems built using the traditional architecture while also advancing the IoT research that will be conducted for use in weather forecasting. Contrary to the traditional IoT design, data pre-processing is carried out between the sensing and network layers in the suggested architectural model. By performing anomaly detection on the data flow generated between the detection layer and the network layer, the study evaluates the data filtering skills of five different algorithms. The resources provided by Google Colab are used to simulate and practice algorithms. The comparison and simulation results are included in the study’s experimental results section. The comparison and simulation results are included in the study’s experimental results section. Data were produced, and a data flow was built using the Arduino UNO R3, BME280, DS1302, Real Time Clock Module, and ESP32 Microcontroller. The produced data stream was immediately categorized using the SVC, Adaboost, LR, NB, and RF algorithms. Prior to reaching the target, algorithms enable real-time classification of incoming data. Using sensitivity, recall, and f1 score, anomaly detection performance is evaluated. This real-time procedure entails isolating the anomaly data from the massive data produced by the IoT detection layer. Algorithms are also contrasted in terms of how quickly they handle data.

For example, when our test results are examined, it is seen that the performance of the RF algorithm is low in terms of execution time in our test results. This situation is important in terms of discovering the right methods for the most accurate weather forecast, as we stated in the purpose of the study. The IoT-based weather forecast system, which is the backbone of the study, and our test results, show that the data obtained from the sensing layer should be clearing from anomalies with an appropriate data preprocessing approach in order to obtain more meaningful and accurate results from IoT-based systems.

## 7. Conclusions

The results we obtained from the model we simulated for IoT-based weather forecasting were examined. It is predicted which algorithms will achieve healthier results at IoT ends. The data pre-processing model we used in our study employs 2907 sensor data points or 30% of the total set of data. In terms of execution time, the RF algorithm comes in fifth place with 52.82 s. In real-time IoT applications, accuracy score results are crucial, and the outcome was 1.0. Examining the confusion matrices reveals that the RF algorithm has a substantial degree of success by correctly identifying 876 anomalous data points as anomalous and correctly identifying 2031 of 2907 true positives as true positives. However, its failure in execution time shows that the algorithm cannot perform data pre-processing properly in IoT systems that will be designed to be used in weather forecast stations. When our experimental results are examined, the SVC algorithm ranks 4th with 46.46 s in terms of execution time. The algorithm uses 30% of the data set, that is, 2907 sensor data points. The accuracy score result was 1.0. When the confusion matrices of the algorithm are examined, it achieves success by predicting 2031 of 2907 data as true positive, similarly, it has obtained correct results about normal and abnormal data by estimating that 876 anomaly data points are also an anomaly. However, due to its failure in execution time, its success in terms of accuracy score has lost its importance.

When the Adaboost algorithm is examined, it is seen that it ranks 3rd in terms of execution time, classification performance, accuracy score, and execution time. When the accuracy score was examined, it showed better performance by obtaining a result of 1.0 as seen in the other four algorithms. The confusion matrix of the algorithm accomplished better results by predicting 2031 of 2907 data points such as RF, SVC, and LR algorithms as true positive, similarly, it predicts 876 anomaly data points to be an anomaly and obtains correct results about normal and anomaly data. When we examined the results of the data pre-processing performance tests that we subjected to the LR algorithm, we saw that the algorithm takes second place in terms of execution time. It also achieves results such as the RF, SVC, and Adaboost algorithms in terms of accuracy score and confusion matrix. In our test results, the execution time of the NB algorithm performed very well compared to the other four algorithms, and took first place. However, 1998 out of the 2907 data points used for in verification and prediction, which is normal data, are predicted by the algorithm as normal data and true positive, with no anomaly. The algorithm predicts 112 data points, which is normal data, as anomalies and false negatives. The algorithm predicted 34 data points with anomalies as normal and false positives, and the algorithm correctly predicts true negatives and 764 data points with anomalies. The overall accuracy of the algorithm is 0.95. When the classification performance accuracy rates and confusion matrices are examined, it achieved considerable success, with 1.91 s in terms of execution time, and the first algorithm lags behind other algorithms in terms of accuracy.

The four algorithms that are closest to one another among the five in the performance test are RF, LR, Adaboost, and SVC. However, NB is regarded as the best method when CPU and execution time are examined as indicators of data processing time. The fact that the NB algorithm outperforms other algorithms in terms of data processing speed is a significant finding because real-time performance and data processing speed are key factors in IoT systems. The NB algorithm’s performance in terms of speed is a key sign that it would work well with IoT-based weather forecasting systems, even though its accuracy is extremely close to that of the other four algorithms. It is therefore challenging to handle big data as a result of the broad use of the internet of things due to the growth in data output through sensors and the data generated through data collection and processing. Structured data is essentially accurate and meaningful information. However, some of the factors that make it challenging for data management to produce relevant data include unnecessary density and faulty data from semi-structured and unstructured data. This paper examines research on IoT-based weather forecasting systems. However, some of the factors that make it challenging for data management to produce relevant data include superfluous density and faulty data from semi-structured and unstructured data.

This paper examines research on IoT-based weather forecasting systems. It covers IoT concepts, technologies, and what IoT means in daily life. Measures to be taken to show meaningful results from weather forecast stations created with IoT technology are being investigated. The approaches that can be developed for the efficient management of sensor data supplied by IoT sources were reviewed, and the performances of various algorithms were tested, as a result of the literature review and simulation done in the study. At the IoT edges, anomaly detection and performance analysis are carried out using machine-learning (ML) algorithms using the 9690 data points collected by the sensors, which include four-dimensional temperature, humidity, pressure and time data. Our findings demonstrate that the application of data filtering techniques at the IoT edge will support rapid and accurate decision-making at weather stations.

## Figures and Tables

**Table 1:** Random forest classification report.

Random Forest Classification				
	Precision	Recall	F1-Score	Support
0	1.00	1.00	1.00	2031
1	1.00	1.00	1.00	876
Accuracy	-	-	1.00	2907
Macro Avg.	1.00	1.00	1.00	2907
Weight Avg.	1.00	1.00	1.00	2907

**Table 2:** Logistic regression classification report.

Logistic Regression Classification				
	Precision	Recall	F1-Score	Support
0	1.00	1.00	1.00	2031
1	1.00	1.00	1.00	876
Accuracy	-	-	1.00	2907
Macro Avg.	1.00	1.00	1.00	2907
Weight Avg.	1.00	1.00	1.00	2907

**Table 3:** Adaboost classification report.

Adaboost Classification				
	Precision	Recall	F1-Score	Support
0	1.00	1.00	1.00	2031
1	1.00	1.00	1.00	876
Accuracy	-	-	1.00	2907
Macro Avg.	1.00	1.00	1.00	2907
Weight Avg.	1.00	1.00	1.00	2907

**Table 4:** Naive Bayes classification report.

Naive Bayes Classification				
	Precision	Recall	F1-Score	Support
0	0.95	0.98	0.96	2031
1	0.96	0.87	0.91	876
Accuracy	-	-	0.95	2907
Macro Avg.	0.95	0.93	0.94	2907
Weight Avg.	0.95	0.95	0.95	2907

**Table 5:** Support vector machine classification report.

Support Vector Machine Classification				
	Precision	Recall	F1-Score	Support
0	1.00	1.00	1.00	2031
1	1.00	1.00	1.00	876
Accuracy	-	-	1.00	2907
Macro Avg.	1.00	1.00	1.00	2907
Weight Avg.	1.00	1.00	1.00	2907

**Table 6:** Accuracy scores report.

	Accuracy Scores
Random Forest	1.0
Logistic Regression	1.0
Support Vector Machine Classification	1.0
Naive Bayes	0.95
Adaboost	1.0

**Table 7:** Comparison of data processing speed.

	Execution Time
Logistic Regression	2.18 s
Naive Bayes	1.91 s
Support Vector Machine Classification	46.46 s
Random Forest	52.82 s
Adaboost	2.19 s

**Table 8:** Random forest confusion matrix.

		Actual Values	
		Positive (1)	Negative (0)
Predicted Values	Positive (1)	2031	0
	Negative (0)	0	876

**Table 9:** Logistic regression confusion matrix.

		Actual Values	
		Positive (1)	Negative (0)
Predicted Values	Positive (1)	2031	0
	Negative (0)	0	876

**Table 10:** Support vector classifier confusion matrix.

		Actual Values	
		Positive (1)	Negative (0)
Predicted Values	Positive (1)	2031	0
	Negative (0)	0	876

**Table 11:** Naive Bayes confusion matrix.

		Actual Values	
		Positive (1)	Negative (0)
Predicted Values	Positive (1)	1998	34
	Negative (0)	112	764

**Table 12:** Adaboost confusion matrix.

		Actual Values	
		Positive (1)	Negative (0)
Predicted Values	Positive (1)	2031	0
	Negative (0)	0	876

## Data Availability

Please email corresponding author to get the dataset.
